# Allele-specific effect of various dietary fatty acids and ETS1 transcription factor on SCD1 expression

**DOI:** 10.1038/s41598-023-50700-5

**Published:** 2024-01-02

**Authors:** Kinga Tibori, Veronika Zámbó, Gabriella Orosz, Péter Szelényi, Farkas Sarnyai, Viola Tamási, Zsolt Rónai, Miklós Csala, Éva Kereszturi

**Affiliations:** Department of Molecular Biology, Semmelweis University, 1085 Budapest, Hungary

**Keywords:** Lipids, Transcription, Gene regulation, Metabolic disorders, Nutrition disorders, Risk factors

## Abstract

Overnutrition and genetic predisposition are major risk factors for various metabolic disorders. Stearoyl-CoA desaturase-1 (SCD1) plays a key role in these conditions by synthesizing unsaturated fatty acids (FAs), thereby promoting fat storage and alleviating lipotoxicity. Expression of SCD1 is influenced by various saturated and cis-unsaturated FAs, but the possible role of dietary trans FAs (TFAs) and *SCD1* promoter polymorphisms in its regulations has not been addressed. Therefore, we aimed to investigate the impact of the two main TFAs, vaccenate and elaidate, and four common promoter polymorphisms (rs1054411, rs670213, rs2275657, rs2275656) on SCD1 expression in HEK293T and HepG2 cell cultures using luciferase reporter assay, qPCR and immunoblotting. We found that SCD1 protein and mRNA levels as well as *SCD1* promoter activity are markedly elevated by elaidate, but not altered by vaccenate. The promoter polymorphisms did not affect the basal transcriptional activity of *SCD1*. However, the minor allele of rs1054411 increased *SCD1* expression in the presence of various FAs. Moreover, this variant was predicted in silico and verified in vitro to reduce the binding of ETS1 transcription factor to *SCD1* promoter. Although we could not confirm an association with type 2 diabetes mellitus, the FA-dependent and ETS1-mediated effect of rs1054411 polymorphism deserves further investigation as it may modulate the development of lipid metabolism-related conditions.

## Introduction

Fatty acids (FAs) are the main building blocks of the structurally very heterogeneous lipid compounds. The diverse functions of these lipids include membrane formation, energy storage and signal transduction^1^. Excessive FA overload leads to abnormal lipid accumulation, cell dysfunction, or even cell death in both adipose and non-adipose tissues^2–4^, a phenomenon known as lipotoxicity. Several studies in various experimental settings suggest that the saturated (SFAs) and the unsaturated FAs (UFAs) contribute differently to lipotoxicity. The deleterious effects of palmitate, which can be mitigated in the presence of oleate, have already been widely demonstrated^5–8^. In contrast, very little is known about the cellular effects of dietary trans fatty acids (TFAs), despite their implication in type 2 diabetes mellitus (T2DM) and cardiovascular diseases^9^, systemic inflammation^10^, dyslipidemia^11^, endothelial dysfunction^12^, different types of cancer^13^ and neurodegenerative disorders^14^. TFAs are UFAs that contain at least one double bond in trans configuration. The vast majority of TFAs are produced in industrial processes (industrial or iTFAs). Elaidate is the primary iTFA, often found in partially hydrogenated vegetable oils. Ruminants' milk and meat also contain small amounts of naturally occurring TFAs (ruminant or rTFAs), mainly the trans isomer of vaccenate^9^.

Human de novo FA synthesis yields palmitate, which can be extended by two-carbon units to longer saturated chains, thus, the balanced production of saturated (SFAs) and mono- (MUFAs) or endogenous polyunsaturated FAs (PUFAs) is reliant on desaturation. Although cis double bonds can be formed at different positions up to 9, the first one must be created at *Δ*9 position by stearoyl-CoA desaturase-1 (SCD1), which makes the activity of SCD1 crucial for the overall desaturation process^15^.

The desaturase activity currently available to the cell is dependent on the level of the SCD1 enzyme, which, in turn, is determined by (*i*) the transcriptional and post-transcriptional regulation of the synthesis and (*ii*) the regulation of the protein degradation. The gene contains an alternative polyadenylation site, which results in two different 3’ untranslated regions (3’ UTRs) and consequently different mRNA transcripts with distinct stability, which may allow a rapid and efficient regulation of protein levels^16^. *SCD1* transcription is also controlled by several activating (insulin, growth factors, glucose, sucrose and cholesterol) and inhibitory (leptin, glucagon, docosahexaenoic acid and arachidonic acid) agents acting through various transcription factors (TFs) (LXR, SREBP, PPAR, C/EBP, TR)^17^. It is evident that intracellular FA supply and composition also modulates SCD1 expression as the transcription of *SCD1* gene is efficiently induced by saturated FAs such as palmitate or stearate, whereas it is repressed by cis-MUFAs (e.g., oleate)^18^. In addition, a highly conserved PUFA-sensitive region (PUFARE) has also been identified in the upstream regulatory region of *SCD1*, which significantly down-regulates the intracellular *SCD1* mRNA pool in the presence of linoleate^18–20^. SCD1 is an enzyme with a short half-life and its intracellular abundance is fine-tuned in the short term by the rapid degradation of the protein. Its N-terminus contains a PEST degradation domain that is presumably involved in targeting the protein to the proteasome during the ERAD process^21^. The stability of SCD1 protein in lung cancer cells is increased by tyrosine phosphorylation^22^, and a UFA-induced degradation of stearoyl-CoA desaturase has been reported in Drosophila, although the latter phenomenon has not been demonstrated in the case of the human orthologue^23^.

Natural genetic variations in *SCD1* may also alter the above-described molecular mechanisms underlying the control of SCD1 expression. A common missense single nucleotide polymorphism (SNP) (rs2234970, M224L) was found to increase protein and mRNA stability, which could be further enhanced by different FAs, and resulted in elevated intracellular UFA levels^24^. Furthermore, the GG haplotype of two intronic *SCD1* variants (rs55710213 and rs56334587) significantly reduced *SCD1* expression by disrupting HNF4A TF binding^25^. Conversely, a SNP (rs41290540) located in the 3’ UTR increased *SCD1* expression in a luciferase reporter assay by truncating a miR-498 target sequence^26^.

Changes in the intracellular level of SCD1 may represent risk factors for the development of various diseases. However, despite their obvious health impact, the possible modulatory effects of either TFAs or natural human polymorphisms in the *SCD1* promoter have not been investigated. In the present study, we aimed to investigate the effect of different saturated, cis- and trans-unsaturated fatty acids on the expression of SCD1 in vitro at mRNA and protein level. In addition, we planned to address the potential role of FAs, in particular elaidate and vaccenate, and that of selected polymorphisms in the 5’ region of *SCD1* in modulating promoter activity, both separately and in combination, in a luciferase reporter system. We also aimed to analyze the potential impact of functional promoter variants in silico and in vitro on TF binding site modification and their correlation with T2DM in an association study.

## Results

### Alteration in SCD1 protein level in response to different dietary FAs

**Figure 1:**
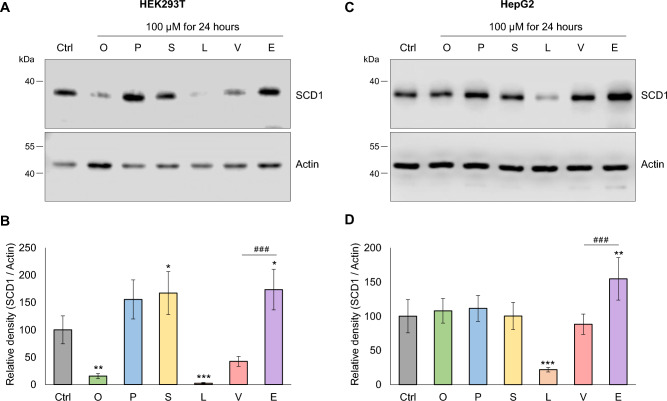
Effect of various dietary FAs on the expression of SCD1 in HEK293T and HepG2 cells. Cells were treated with BSA-conjugated oleate (O), palmitate (P), stearate (S), linoleate (L), vaccenate (V) or elaidate (E) at a final concentration of 100 μM for 24 h. Immunoblot analysis of cell lysates (20 µg protein per lane) was carried out using anti-SCD1 and anti-Actin antibodies. Representative results of four (HEK293T) or five (HepG2) independent experiments are shown (**A**, **C**). Uncropped versions of all parallel blot images are available in the Supplementary Information file. The band intensities were determined by densitometry and SCD1/Actin ratios are shown as bar graphs (**B**, **D**). Statistical analysis was performed with the Tukey–Kramer Multiple Comparisons Test. Data are shown as mean values ± SD. Ctrl: control; *p < 0.05; **p < 0.01; *** and ^###^*p* < 0.001.

The modulating effect of the dietary SFAs, cis-MUFAs and PUFAs on the expression of the main desaturase enzyme, SCD1 has been well characterized^18–20^, however the possible regulatory impact of the two major dietary TFAs, i.e., elaidate (18:1 trans-*Δ*9) of industrial origin and the naturally occurring vaccenate (18:1 trans-*Δ*11) remains to be elucidated. To compare the effects of TFAs with those of other FAs on the cellular level of the SCD1 protein, HEK293T and HepG2 cells were treated with BSA-conjugated oleate, palmitate, stearate, linoleate, vaccenate and elaidate at a final concentration of 100 µM for 24 h and their SCD1 content was assessed by immunoblotting (Fig. 1A,C) then evaluated by densitometry (Fig. 1B,D). Consistent with the literature, monounsaturated oleate and polyunsaturated linoleate treatment resulted in significantly lower intracellular protein levels in HEK293T cells (Fig. 1A). Oleate reduced the amount of the desaturase enzyme to less than a fifth of the control level in untreated cells, and SCD1 level was barely detectable in the cells treated with linoleate (Fig. 1B). Consistent with previous studies, a slight increase in SCD1 protein levels was observed in response to saturated palmitate and stearate, although the change was only statistically significant in the latter (Fig. 1B). Most importantly, both TFAs tested (elaidate and vaccenate) markedly affected SCD1 protein amounts in HEK293T cells, but in opposite directions. Since SCD1 levels were approximately halved by vaccinate and almost doubled by elaidate, the opposite impact of the two TFAs resulted in a significant difference of more than fourfold between the SCD1 protein content detected in cells treated with the two TFAs (Fig. 1A,B).

The impact of the same FAs on SCD1 protein levels was also tested in HepG2 cells, which are of hepatocyte origin and thus more relevant for lipid metabolism. Although intracellular SCD1 content was not significantly altered upon administration of oleate or SFAs, the repression by linoleate, the induction by elaidate and the significant difference between the two TFAs could also be demonstrated in HepG2 cells at the protein level (Fig. 1C,D).

### Effect of FAs on *SCD1* expression at the mRNA level

**Figure 2:**
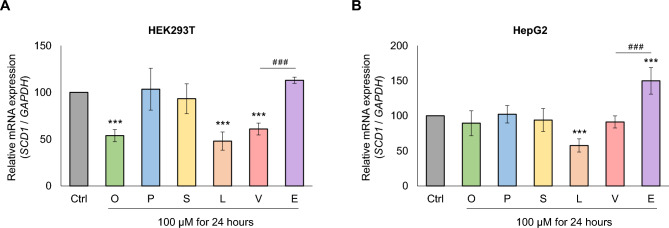
*SCD1* mRNA expression in FA-treated HEK293T and HepG2 cells. The levels of endogenous *SCD1* mRNA were measured in HEK293T (**A**) and HepG2 (**B**) cells treated with different FAs. FA treatment and sample preparation were performed as described in *Materials and Methods*. qPCR was carried out using *SCD1* and *GAPDH* sequence specific primers as indicated in *Materials and Methods*. The diagram presented depicts the results of six independent measurements. Statistical analysis was performed with the Tukey–Kramer Multiple Comparisons Test. Data are shown as mean values ± S.D. *** or ^###^*p* < 0.001.

The observed impact of the FAs tested on intracellular SCD1 protein levels may be attributed to changes in transcriptional activity, mRNA stability or protein stability. To learn more about the underlying mechanism, *SCD1* expression was also studied at mRNA levels in HEK293T and HepG2 cells. After FA treatment, the gDNA-free total RNA extracts of the cells were reverse transcribed into cDNA and mRNA expression was assessed by qPCR as described in *Materials and Methods* section. Consistent with the changes in protein expression, the *SCD1* mRNA content showed a very similar pattern after administration of dietary FAs (Fig. 2A,B). In the HEK293T cell line, oleate, linoleate and vaccenate significantly decreased the expression of *SCD1* mRNA, whereas palmitate, stearate and elaidate did not cause any measurable change. Again, the two TFAs resulted in remarkably different expression levels, i.e., the amount of *SCD1* mRNA was approximately twice as high in elaidate-treated cells than in vaccenate-treated ones (Fig. 2A). The expression pattern of *SCD1* mRNA in HepG2 cells also faithfully reflected the protein levels detected by immunoblotting, so both the down- and up-regulating effects of linoleate and elaidate, respectively, were seen on *SCD1* mRNA quantities. The mRNA levels in the elaidate-treated samples exceeded those in the vaccenate-treated ones to a similar extent as observed in HEK293T cells (Fig. 2B).

### Effect of FAs through *SCD1* promoter in a luciferase reporter system

**Figure 3:**
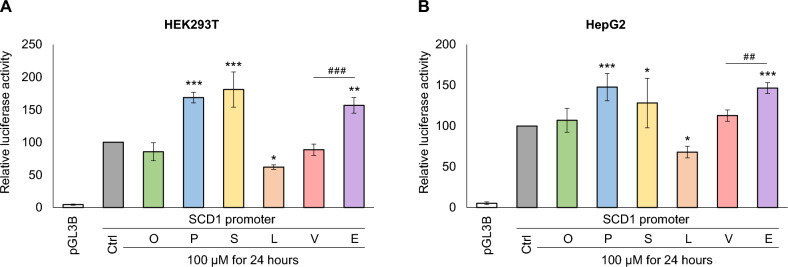
Effect of dietary FAs on *SCD1* promoter activity. Transient transfection and FA treatment of HEK293T (**A**) and HepG2 (**B**) cells were performed as described in *Materials and Methods*. pCMV-*β*-gal vector served as transfection control. Luciferase and *β*-galactosidase enzyme activities were measured as indicated in *Materials and Methods* and their relative ratios are shown as bar graphs. The diagram depicts the results of three (HEK293T) or six (HepG2) independent measurements normalized to pGL3-SCD1 promoter vector. Data are shown as mean values ± S.D. Statistical analysis was performed by using the Tukey–Kramer Multiple Comparisons Test. Ctrl: control; O: oleate; P: palmitate; S: stearate; L: linoleate; V: vaccenate; E: elaidate; *p < 0.05; **p < 0.01; *** and ^###^*p* < 0.001.

**Figure 4:**
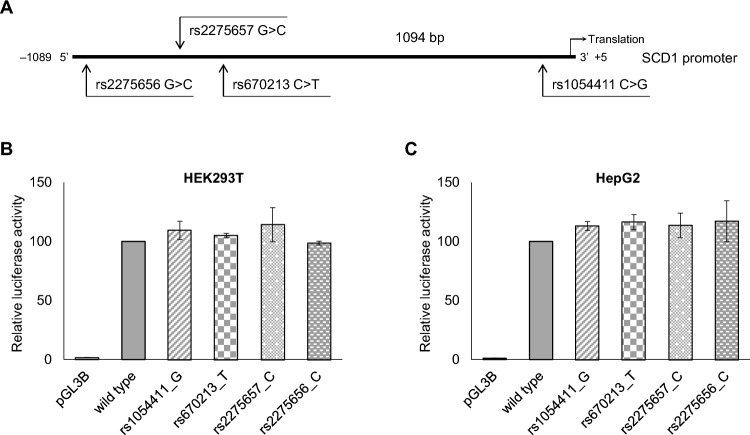
Position (**A**) and effect of *SCD1* promoter SNPs on relative luciferase activity in HEK293T (**B**) and HepG2 (**C**) cells. The subcloned region of *SCD1* promoter, the translational start site and the location, allelic options and ID number of the four selected polymorphisms are presented. Transfection was performed as described in *Materials and Methods*. pCMV-*β*-gal vector served as transfection control. Luciferase and *β*-galactosidase enzyme activities were measured as indicated in *Materials and Methods* and their relative ratios are shown as bar graphs. The diagram depicts the results of three independent measurements normalized to the wild type *SCD1* promoter. Data are shown as mean values ± S.D. Statistical analysis was performed by using the Tukey–Kramer Multiple Comparisons Test.

It became evident that, similarly to oleate, linoleate, palmitate and stearate^18–20^, TFAs also remarkably affect the expression of SCD1. We wanted to investigate whether this effect is based on transcriptional regulation and/or RNA stabilization. To this end, the 1094 base pair long section of the 5’ regulatory region of *SCD1* (Fig. 4A) was cloned into pGL3-Basic vector and used in a luciferase reporter system to assess the *SCD1* promoter-dependent transcriptional effects. HEK293T and HepG2 cells transfected with pGL3-SCD1 promoter construct were treated with BSA-conjugated oleate, palmitate, stearate, linoleate, vaccenate or elaidate at 100 µM concentration for 24 h. After harvesting the cells, relative luciferase and *β*-galactosidase activities were determined. The subcloned *SCD1* 5’ regulatory region worked as a potent promoter, increasing the relative luciferase activity in both cell lines by approximately 20-fold compared to pGL3-B (Fig. 3). As expected, a significant suppression by linoleate on luciferase activity was seen in both cell lines, but surprisingly, no such phenomenon was observed for oleate. The two SFAs significantly enhanced the relative luciferase activity in both HEK293T (Fig. 3A) and HepG2 (Fig. 3B) cells. Of the two TFAs, elaidate caused an approximately 50% increase in *SCD1* promoter activity, whereas vaccenate was not effective in this assessment, thus the significant difference between the two TFAs was also revealed at the transcriptional regulation of *SCD1*.

### Modulation of FA-dependent control of *SCD1* expression by promoter SNPs

Regulation of the transcription can be significantly affected by genetic variations of the promoter region. Therefore, we tested the known SNPs in the *SCD1* promoter with minor allele frequencies (MAFs) above 5% for their potential impact on FA-sensitivity of *SCD1* expression. Using the NCBI dbSNP and Ensembl databases, we selected four polymorphisms that met the above criterion. Identification number, position, allelic variants and MAF value of these variants are presented in Supplementary Table S1 and Fig. 4A. All four SNPs were flagged as modifiers by the VEP prediction program, indicating their potential functionality (Supplementary Table S1), yet neither of them has been experimentally investigated. We generated both alleles of these genetic variants in the pGL3-SCD1 promoter vector by site-directed mutagenesis and analyzed them by a luciferase reporter assay in HEK293T and HepG2 cells after transient transfection. This in vitro approach revealed no significant modulation of basal promoter activity by any of the four polymorphisms, i.e., none of the minor allelic variants altered the relative luciferase activity compared to the major variant (hereafter referred to as wild type) in either of the two human cell lines (Fig. 4B.C).

**Figure 5:**
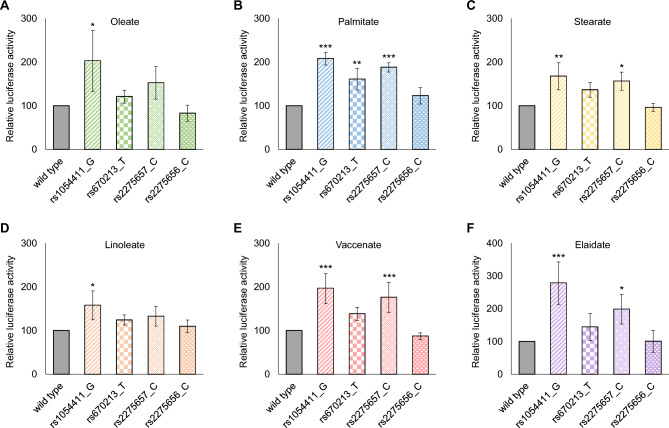
Modulating effect of promoter polymorphisms on *SCD1* promoter activity in the presence of various FAs in HEK293T cells. Transient transfection and FA treatment of HEK293T cells were performed as described in *Materials and Methods*. pCMV-*β*-gal vector served as transfection control. Luciferase and *β*-galactosidase enzyme activities were measured as indicated in *Materials and Methods* and their relative ratios are shown as bar graphs. The diagram depicts the results of at least three independent measurements normalized to the relative luciferase activity of oleate- (**A**), palmitate- (**B**), stearate- (**C**), linoleate- (**D**), vaccenate- (**E**) or elaidate-treated (**F**) wild type *SCD1* promoter containing reporter vector. Data are shown as mean values ± S.D. Statistical analysis was performed by using the Tukey–Kramer Multiple Comparisons Test. O: oleate; P: palmitate; S: stearate; L: linoleate; V: vaccenate; E: elaidate; *p < 0.05; **p < 0.01; *** *p* < 0.001.

To further analyze the potential effect of these polymorphisms, the promoter activity of the allelic variants was also tested in the presence of oleate, palmitate, stearate, linoleate, vaccenate or elaidate. HEK293T cells were treated with 100 µM BSA-conjugated FAs 5 h after transient transfection for 24 h, and the relative luciferase enzyme activities were measured in the collected samples as described in *Materials and Methods*. The G allele of the rs1054411 SNP, which was shown to have the same basal promoter activity as the wild type (see Fig. 4B,C), resulted in a significantly higher promoter activity than the wild type when exposed to any of the six FAs (Fig. 5). It is noteworthy that the most pronounced, almost threefold increase in the relative luciferase activity compared to the wild type was observed upon elaidate treatment (Fig. 5F). When the same allelic variant was tested in HepG2 cells, the allele-specific impact of some other FAs was also observed, i.e., the relative luciferase activity with the rs1054411_G promoter was increased not only by elaidate but also by linoleate and vaccenate in the reporter system (Supplementary Fig. S1).

Beside the general FA-dependent enhancement of rs1054411_G promoter activity, it is also intriguing that the rs2275656_C variant apparently lost its FA-sensitivity, as it did not respond to any FA treatments investigated (Fig. 5). The other two tested *SCD1* promoter variants (rs670213_T and rs2275657_C) showed rather diverse responsiveness to FAs. While neither of them modified the effect of the cis-unsaturated FAs (oleate and linoleate) compared to the wild type (Fig. 5A,D), rs2275657_C showed a significantly increased promoter activity both in the presence of SFAs (palmitate and stearate) (Fig. 5B,C) and TFAs (vaccenate and elaidate) (Fig. 5E,F), and such modulation was only observed with palmitate in case of rs670213_T (Fig. 5B).

### In silico prediction of the effect of *SCD1* promoter SNPs on TF binding

**Figure 6:**
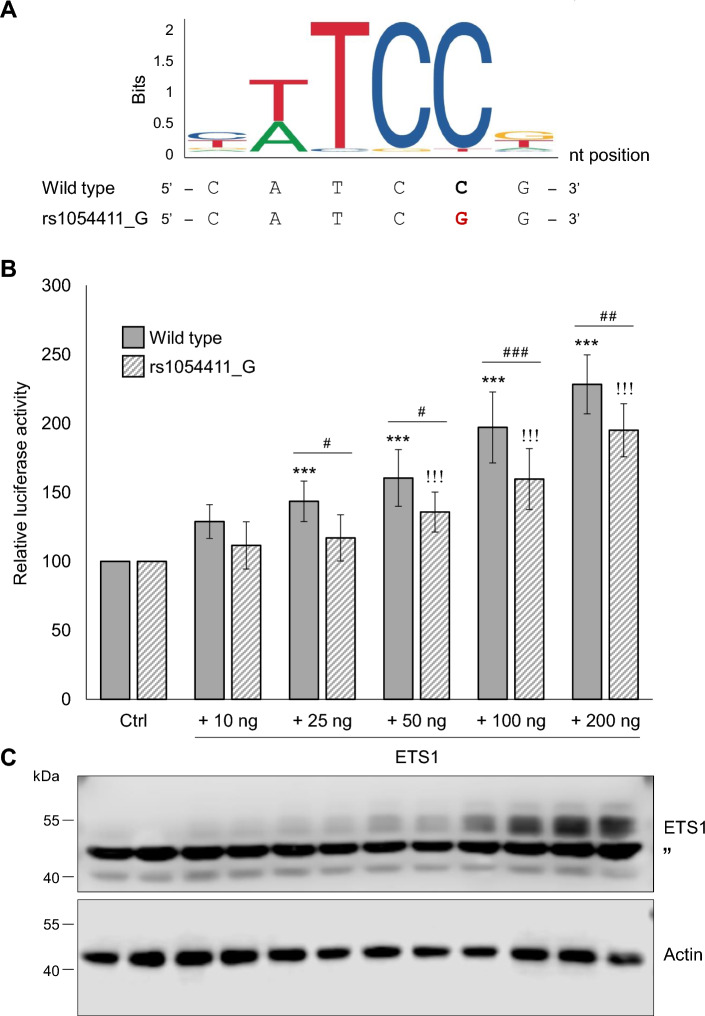
Effect of rs1054411 SNP on the ETS1-mediated stimulation of *SCD1* promoter activity in a luciferase reporter system. (**A**) Structure of the ETS1 TF binding sequence modified by rs1054411_G is illustrated. The polymorphic site is highlighted in bold and red. (**B**) Transient co-transfection of HEK293T cells was performed as described in *Materials and Methods*. pCMV-*β*-gal vector served as transfection control. Luciferase and *β*-galactosidase enzyme activities were measured as indicated in *Materials and Methods* and their relative ratios are shown as bar graphs. The diagram depicts the results of three to twelve independent measurements normalized to ETS1-free wild type or rs1054411_G pGL3-SCD1 promoter vector, respectively. Data are shown as mean values ± S.D. Statistical analysis was performed by using the Tukey–Kramer Multiple Comparisons Test. Ctrl: control; ^#^*p* < 0.05; ^##^*p* < 0.01; ***, ^###^ or ^!!!^*p* < 0.001. **C** The increasing amount of ETS1 protein expressed in HEK293T cells co-transfected with an increasing amount of *ETS1* plasmid (10, 25, 50, 100 or 200 ng) was verified by immunoblotting. Immunoblot analysis of cell lysates (20 µg protein per lane) was carried out using anti-ETS1 and anti-Actin antibodies as described in *Materials and Methods*. Uncropped versions of all parallel blot images are available in the Supplementary Information file. Ctrl: control; ” indicates non-specific band on ETS1 immunoblot; ^#^*p* < 0.05; ^##^*p* < 0.01; ***, ^###^ or ^!!!^*p* < 0.001.

The possible impact of the four SNPs on TF binding sites in the promoter of the *SCD1* gene was analyzed in silico using the JASPAR transcription factor binding site prediction program. Specifically, we addressed the question whether the alteration of the four nucleotides affected by the polymorphisms could cause a predictable change in the binding probability of any TFs in this region. The in silico TF selection protocol has been published previously^27^. Briefly, for each SNP, the two 41-nucleotide long DNA segments were compared, in which the polymorphic nucleotide was located at position 21. Hits were selected with a relative TF binding score greater than 80% for at least one allele and a relative score difference of at least 15% between the two alleles. Five predicted TF binding sites for rs1054411, two for rs670213 and rs2275657, and seven for rs2275656 met the above criteria, their IDs and relative binding probabilities are summarized in Table 1 and Supplementary Table S2. The predicted effect of the rs1054411 polymorphism on ETS1 TF binding appeared to be the most probable hit in the screen. ETS1 had the highest binding probability score of all hits for the wild type sequence (rs1054411_C: 98.14%), and also the largest difference between the two alleles, with the minor allele (rs1054411_G) 21.6% less likely to form an ETS1 TF binding site (Table 1). The predicted large difference between the two alleles of this SNP is not surprising, considering that the polymorphic nucleotide is located at the fifth, highly conserved position of the six-nucleotide long consensus sequence of ETS1 response element (Fig. 6A).

**Table 1:** List of transcription factors that are affected by rs1054411 polymorphism.

Name	TF ID	Strand	Relative score (%)		
			C allele	G allele	Difference
NFATC3	MA0625.2	+	61.45	80.39	18.94
SOX18	MA1563.1	‒	65.39	81.67	16.28
SPI1	MA0080.1	‒	81.85	66.40	‒15.45
ETV5	MA0765.1	‒	85.96	69.13	‒16.83
ETS1	MA0098.1	+	98.14	76.54	‒21.60

Positive or negative values of the relative score differences indicate that the minor allele increases or decreases the probability of TF binding, respectively.

### Allele-specific but not FA-dependent impact of ETS1 on *SCD1* promoter activity

In light of the FA sensitivity of the rs1054411_G allele variant observed in our previous experiments (see Fig. 5), and the highly diverse regulatory mechanism of ETS1^28^, the question was raised whether the ETS1 expression is also FA sensitive. To address this question, HEK293T cells were treated for 24 h with 100 µM BSA-conjugated oleate, palmitate, stearate, linoleate, vaccenate or elaidate, and the endogenous gene expression of *ETS1* was assessed in the collected samples, but the *ETS1* mRNA levels were not modified by any of the FAs tested (Supplementary Fig. S2). The possible FA-dependent modification of endogenous ETS1 protein levels could not be examined because the low sensitivity of the commercially available ETS1 antibodies did not allow for proper detection of the protein without overexpression. The amount of ETS1 protein overexpressed in transfected cells was not affected by the presence of FAs (Supplementary Fig. S2).

Since the ETS1 binding was predicted to be affected by the rs1054411 polymorphism, the allele-specific effect of this TF was tested in a luciferase reporter system in vitro (Fig. 6). HEK293T cells were transiently co-transfected with the pGL3-SCD1 promoter plasmid containing either the wild type or the rs1054411_G variant, together with different amounts of ETS1 expression vector. 24 h after transfection, the cells were harvested, and the relative luciferase activities were measured. The samples of the co-transfected cells were compared to the corresponding wild type or rs1054411_G promoter activities without ETS1 overexpression. ETS1 overexpression resulted in enhanced activities of both versions of the promoter, and the effect was growing in parallel with the increasing amounts of ETS1 expression construct applied (Fig. 6B) and with the increasing amount of ETS1 protein, as verified by immunoblotting (Fig. 6C). However, there was a marked difference between the extent of the ETS1-dependent enhancement between the two promoter versions tested. In case of the wild type *SCD1* promoter, even the small amount of ETS1 protein yielded by 25 ng plasmid significantly increased the relative luciferase activity by almost one and a half times (Fig. 6B). Furthermore, the largest amount of ETS1 plasmid applied (200 ng) resulted in more than a twofold increase in the activity of the wild type *SCD1* promoter. Although the activity of the promoter holding the G allele of rs1054411 SNP showed an increasing trend with increasing ETS1 levels, it remained below that of the wild type in all experimental conditions (Fig. 6B), which finding is in agreement with the in silico predicted reduction of the TF binding capacity (see Table 1). The difference between the activity of the two alleles of rs1054411 after co-transfection with 25, 50, 100 or 200 ng ETS1 plasmid was significant in favor of the wild type sequence (Fig. 6B).

To further analyze the possible interaction between ETS1 TF and the presence of FAs, a combination of co-transfection and FA treatment was performed. For this experiment elaidate was selected, as the rs1054411_G allele in its presence was the most potent in enhancing *SCD1* promoter activity. HEK293T cells were transiently transfected with wild type or rs1054411_G allele-containing *SCD1* promoter construct with or without 100 ng ETS1 expression vector and/or in the presence or absence of 100 µM BSA-conjugated elaidate (Supplementary Fig. S3). The appropriate amount of ETS1 vector and the optimal concentration of elaidate were chosen based on our experiments shown in Figs. 5 and 6. Samples were collected 24 h after FA treatment and relative luciferase activities were measured. As expected, ETS1 inhibited and elaidate enhanced *SCD1* promoter activity in the presence of the rs1054411_G allele. When the two agents were administered together, however, the negative effect of ETS1 could neither reverse nor neutralize the enhancing effect of elaidate on rs1054411_G allele-specific promoter activity, but merely reduced it by about half (73% vs 37%, Supplementary Fig. S3).

### Association analysis between rs1054411 SNP and T2DM

The possible association between the rs1054411 polymorphism and T2DM was investigated in a case–control setup. The results are summarized in Table 2. The observed genotype distribution in the control group was in agreement with the expected values based on the Hardy–Weinberg equilibrium (*χ*^2^-test *p* = 0.911). Allele frequencies were in line with the European population data available in 1000Genomes (MAF: 41 vs. 40%), however, in our control group the minor allele was slightly overrepresented compared to both ALFA (MAF: 35%) and global frequencies (MAF: 28%). Association analyses were performed using both allele- and genotype-based approaches, including the dominant model (i.e., genotype combination). As shown in Table 2, the frequency of the G allele was slightly but not significantly lower in the T2DM group in all comparisons. Due to the limited number of samples that could be included in the study, the power was as low as 35.6% suggesting that the lack of statistically significant result does not exclude the putative role of the SNP in the genetic risk of T2DM.

**Table 2:** Comparison of allele, genotype, and genotype combination frequencies of rs1054411 polymorphism in control and T2DM groups.

	Control (N = 370)		T2DM (N = 282)	
	N	%	N	%
Allele				
C	437	59	351	62
G	303	41	213	38
* χ*^2^	*p* = 0.2447			
Genotype				
C/C	127	34	107	38
C/G	183	49	137	49
G/G	60	16	38	13
* χ*^2^	*p* = 0.4943			
Genotype combination				
C +	310	84	244	87
C‒	60	16	38	13
* χ*^2^	*p = 0.3319*			

## Discussion

The role of SFAs and cis-unsaturated FAs in regulating the expression of SCD1, one of the key enzymes of lipid metabolism, is a much-researched topic in the literature. SFAs, not surprisingly and in line with our results (Figs. 1, 2, 3), tend to increase the amount of SCD1 available in the cell, thus enhancing their own conversion to unsaturated FAs, which favors their utilization in lipid synthesis and hence mitigates their own lipotoxic effects throughout the body^29,30^. The attenuating effect of cis-unsaturated FAs on SCD1 expression is also well known^20,31^, however, the mechanism of action of monounsaturated oleate and polyunsaturated linoleate may slightly differ. Linoleate is thought to interfere with desaturation through the regulation of transcription, and it clearly repressed SCD1 expression at all three levels we examined (promoter activity, mRNA and protein levels) in both cell lines. This is in agreement with the fact that a PUFA-responsive element has been described and characterized in the upstream regulatory region of both human and mouse *SCD1* genes^18–20^. While several studies have demonstrated the SCD1-repressing effect of oleate in a variety of ways^32^, the exact mechanism is still unknown. Although oleate clearly decreases the desaturase the level of the enzyme and thus the desaturase activity, there are inconsistent findings with respect to the effect at the mRNA level, and even more so with respect to the promoter activity, suggesting that oleate acts through mRNA and/or protein stabilization rather than reducing transcription^33^. It should be noted that the well-characterized reducing effect of oleate on SCD1 was not seen at all three regulatory levels we examined in HepG2 cells, and the effect of other FAs was also rather modest in this cell line, probably due to the relatively high FA tolerance of HepG2 cells^34^.

Human studies have reported a positive correlation between TFA intake and the development of lipid metabolism-related conditions such as the metabolic syndrome, T2DM, cardiovascular disease and cancer^9^, however, the potential role of TFAs in the regulation of FA desaturation has been so far neglected. It has been reported that TFAs may have similar protective effects against palmitate toxicity as cis-unsaturated oleate in cell cultures^6^. They promote inflammation and ER stress to a much lesser extent than the most lipotoxicity-inducing SFAs, so they are currently considered less harmful^35–37^. TFAs were also reported to stimulate cholesterol synthesis in vitro^38^ and enhance hepatic fat accumulation in vivo^39^. It is therefore clear that the TFAs have special effects on the metabolism and basic physiological processes of the human body, that are different from other dietary FAs, and there is an ongoing scientific debate about the similarities and differences between iTFAs and rTFAs in terms of their health impacts. Several human studies have reported that the adverse effects of TFAs are limited to iTFAs^40,41^, while rTFAs have been shown to be harmless or even beneficial for metabolic health^42,43^. In contrast, other epidemiological and clinical studies have shown that rTFAs are as culpable as iTFAs in the development of metabolic and cardiovascular diseases^44,45^. In the light of the controversial data, we considered it important to investigate and compare the effect of iTFAs and rTFAs on the expression of SCD1, as modulating the amount of this key enzyme of lipid metabolism would provide an obvious means for the TFAs to influence lipid homeostasis in the human body. It was previously published that the administration of elaidate increased the desaturation index in HASMC, HUVEC and HepG2 cells^31,46^, as well as the *SCD1* mRNA expression in trophoblast and HASMC cells^31,47^, while vaccenate did not seem to alter FA desaturation^31,46,48^. To refine the overall picture, we systematically examined the effect of elaidate and vaccenate on SCD1 at the protein and mRNA levels, as well as on the promoter activity in a luciferase reporter system in HEK293T and HepG2 cell lines. Consistent with the limited scientific data summarized above, a significant difference was detected between the two types of TFAs, as a marked inducing effect of elaidate was detected in all the three investigated parameters and in both cell lines, which contrasted with the neutral nature of vaccenate (Figs. 1, 2, 3).

Studying FA-dependent regulation of SCD1 expression is of particular interest since consequential alterations in lipid desaturation have been implicated in a variety of diseases. Elevated cellular activity of SCD1, an enzyme catalyzing the rate-determining step of FA desaturation, which in turn is essential for major synthetic pathways of lipid metabolism, significantly increases the likelihood of developing obesity and related conditions such as the metabolic syndrome, diabetes, insulin resistance and hepatic steatosis^17^. SCD1 has also been identified as an important modulator of cancer cell survival and progression^49^, and its expression is associated with poor prognosis in several cancer types^50^. Genetic polymorphisms may also regulate the intracellular availability of a gene or protein in the context of gene-environment interactions, independently of, or possibly in combination with, the effects of FAs^51^. As SNPs in the upstream regulatory region of *SCD1* have not yet been functionally investigated, and only the rs670213 polymorphism has been analyzed and found to be unrelated to metabolic risk^52,53^, in the present study, we tested the promoter polymorphisms in vitro in a luciferase reporter system both in the absence (Fig. 4) and presence (Fig. 5, Supplementary Fig. S1) of various dietary FAs. The observed allele-specific inducing properties of the FAs are not without precedent, as the elevated expression of the only common missense *SCD1* variant (rs2234970) is also attributed partly to a FA-mediated and sequence-dependent protein stabilization^24^. Although the rs1054411 SNP, which was found to be functional in the presence of FAs (Fig. 5), did not show significant association with T2DM in our study (Table 2), its role in the development of metabolic conditions cannot be ruled out completely. In light of the results of our in silico analysis and in vitro experiments, its possible correlation with diabetes should be assessed in larger samples, complemented with other phenotypic and clinical data (e.g., dietary intake composition and serum FA profile). The NCBI LDmatrix tool indicates complete linkage disequilibrium for rs2275656 and rs2275657 SNPs, while the other two loci are not or only partially linked. In line with this, the NCBI LDhap predicts the presence of five haplotypes out of 16 possible combinations of the four SNPs in the European population. This suggests that the rs670213 and rs1054411 SNPs are evolutionally younger, their polymorphic alleles are likely to have arisen and combined with the GG haplotype of rs2275656 and rs2275657, whereas the CC haplotype of rs2275656 and rs2275657 is only found with the ancestral alleles (both C) of rs670213 and rs1054411. Taking these together, it may be worthwhile to analyze the haplotypes of the four *SCD1* promoter SNPs from both a functional and an association perspective in the future.

ETS1, a member of the ETS protein family of TFs, regulates the expression of a diverse set of proteins through its interaction with specific consensus sequences upstream of target genes. Increased expression of ETS1 has been detected in a wide variety of cancers and associated particularly with tumor progression and invasion, and there is also increasing interest in its role in basic metabolic processes^54^, as it has been revealed to up-regulate key enzymes in FA metabolism^55^. Although the highly diversified transcriptional, post-transcriptional and post-translational control of ETS1 has been thoroughly characterized^28^, the possible role of FAs in this regulation has not been investigated. Although ETS1 expression itself was not found to be FA-sensitive in our experimental setup (Supplementary Fig. S2), our in silico analysis identified ETS1 as a TF with allele-specific binding to the *SCD1* promoter region carrying the rs1054411 SNP (Table 1). Moreover, the predicted allele-specific binding of ETS1 was also verified in vitro (Fig. 6).

In summary, our results indicate that the two most common TFAs, industrial elaidate and natural vaccenate, have significantly different effects on SCD1 expression, as the induction by elaidate manifested in vitro not only at the protein and mRNA levels of the endogenous expression but also at the promoter activity assessed in a reporter gene model. Among the investigated promoter polymorphisms, the rs1054411, which did not modify basal *SCD1* expression, largely affected *SCD1* promoter activity in the presence of different dietary FAs or under the influence of ETS1 TF, as measured by using a luciferase reporter assay.

Elevation of SCD1 expression in various health conditions is of utmost importance, whether as a cause or a consequence^17,32^. The enzyme is a promising target for the treatment of metabolic diseases, and efforts have been made to develop liver-targeted SCD1 inhibitors^56^. Since genetic variations have a major impact on the efficacy of therapy^57,58^, *SCD1* variants, including functional promoter polymorphisms, such as rs1054411, are likely to alter the effectiveness or even the need for medical treatment with SCD1 inhibitors. The development of individualized therapeutic protocols based on genetic profiling seems a reasonable future goal in the treatment of lipid metabolism-related diseases. However, this goal can only be achieved if the pathomechanisms are understood at the level of gene-environment interaction, which requires both detailed functional characterization of disease-associated gene polymorphisms and thorough mapping of environmental risk factors.

The main strength of this work lies in its diversity, as the opposing effects of the two trans-monounsaturated FAs on SCD1 have been successfully demonstrated at multiple levels and in different cell lines. Furthermore, a unique FA-dependent transcriptional modulation mechanism of the rs1054411 SNP in the *SCD1* promoter has been identified, which may be further fine-tuned in an allele-specific manner by the ETS1 proto-oncogene TF. However, the association study performed is of limited value as it has very low statistical power due to the rather small sample size. In addition, functional analysis of the four promoter polymorphisms in haplotypes and extension of the in vitro studies to animal models could further increase the reliability of the present work in the future.

## Materials and methods

### Chemicals and materials

Culture medium and supplements were purchased from Thermo Fisher Scientific (Waltham, MA, USA). Oleate, palmitate, stearate, linoleate, elaidate, vaccenate, bovine serum albumin, HEK293T and HepG2 cells were purchased from Sigma-Aldrich (St. Louis, MO, USA). All chemicals used in the study were of analytical grade. All experiments and measurements were performed using Millipore ultrapure water.

### Web-based and own-designed tools for in silico analysis

Based on the NCBI and Ensembl databases, *SCD1* promoter SNPs with MAF above 5% and heterozygosity above 0.095 were selected. The JASPAR (http://jaspar.genereg.net/, accessed on 30 June 2022) open-access, non-redundant TF biding profile database was used to predict the potential effect of rs1054411, rs670213, rs2275657 and rs2275656 polymorphisms on TF binding to the *SCD1* promoter^59^. The allele-specific effect on TF binding was analyzed as previously described^27^. Briefly, both allelic variants of each SNP were compared pairwise. TFs showing a score difference of at least 15% between the two variations of the given polymorphism, and a relative score above 80% for at least one of the alleles, were retained for further analysis. The impact of the selected sequence variants was predicted in silico using the Variant Effect Predictor (https://www.ensembl.org/Homo_sapiens/Tools/VEP/, accessed on 12 January 2023)^60^.

### Plasmid Construction and Mutagenesis

A 1094 base pair fragment of the upstream regulatory region of *SCD1* was amplified from human genomic DNA template by iProof™ High-Fidelity DNA Polymerase (Bio-Rad, Hercules, CA, USA) and cloned into the pGL3-Basic plasmid (pGL3B, Promega, Madison, WI, USA) between the *Xho* I and *Hind* III restriction endonuclease recognition sites with 5’‒AAA TTT **CTC GAG** CAA AAC ATC CCG CAC GCA T–3’ sense and 5’‒AAA TTT **AAG CTT** GGC ATC TTG GCT CTC GGA TG –3’ antisense primers. Bold letters indicate the recognition sites of the two endonucleases, respectively. After purification and restriction endonuclease (Thermo Fisher Scientific, Waltham, MA, USA) digestion, the amplicons were ligated (T4 Ligase, Thermo Fisher Scientific, Waltham, MA, USA) into pGL3B vector (Promega, Madison, WI, USA) upstream the luciferase reporter gene. The natural variants were generated using Q5® Site-Directed Mutagenesis Kit (New England BioLabs, Ipswich, MA, USA) following the manufacturer’s instruction. Mutagenic primers were designed using the online NEB primer design software, NEBaseChanger™. After digestion of the original non-mutated and methylated plasmid by KLD reaction, an aliquot of the constructs was transformed into XL10-Gold® Ultracompetent Cells (Agilent, Santa Clara, CA, USA), which were then screened for positive colonies by PCR. The cloning and mutagenic primers are listed in Supplementary Table S3. The ETS1 expression plasmid was purchased from BioCat (Heidelberg, Germany) with pcDNA3.1(‒) vectorial background. All constructs were verified by Sanger sequencing.

### Cell culture and Transfection

Human embryonic kidney (HEK293T) and hepatocellular carcinoma (HepG2) cells were cultured in 12-well plates (1 × 10^6^ cells per well) in Dulbecco’s modified Eagle medium (DMEM) supplemented with 10% fetal bovine serum and 1% penicillin/streptomycin solution at 37 °C in a humidified atmosphere containing 5% CO_2_. Cells were transfected with 0.5 μg pGL3B-SCD1 promoter constructs using 3 µL Lipofectamine 3000 that was supplemented with 2 µL P3000 (Invitrogen, Carlsbad, CA, USA) in 1 mL DMEM. As a transfection control, 0.5 µg pCMV-*β*-gal plasmid was co-transfected. Cells were harvested and processed 24–30 h after transfection.

### Cell treatment

Oleate, palmitate, stearate, linoleate, elaidate, and vaccenate were diluted in ethanol (Molar Chemicals, Halásztelek, Hungary) to a final concentration of 50 mM and conjugated with 20% FA-free BSA in 1:4 ratio at 50 °C for 1 h. The working solution for FA treatments was prepared freshly in FBS-free and antibiotic-free medium at 100 µM final concentration. The FA treatment was carried out for 24 h in 12-well plates. For luciferase assay, the culture medium was replaced 5 h after transfection and the cells were incubated for a further 24 h.

### Preparation of cell lysates

Cell lysates were prepared for immunoblot analysis by removing the medium and washing the cells twice with PBS. 100 µL RIPA lysis buffer (0.1% SDS, 5 mM EDTA, 150 mM NaCl, 50 mM Tris, 1% Tween 20, 1 mM Na_3_VO_4_, 1 mM PMSF, 10 mM benzamidine, 20 mM NaF, 1 mM pNPP, and protease inhibitor cocktail) was added to each well and the cells were scraped and briefly vortexed. After 15 min incubation at room temperature, the lysates were centrifuged for 5 min at maximum speed in a benchtop centrifuge at 4 °C to remove cell debris. Protein concentration of the supernatant was measured with Pierce® BCA Protein Assay Kit (Thermo Fisher Scientific, Waltham, MA, USA) and the samples were stored at ‒20 °C until further analysis.

For the luciferase reporter assay, cells were washed twice with PBS and then scraped in 100 µL reporter lysis buffer (Promega, Madison, WI, USA) and vortexed briefly. A single freeze–thaw cycle was followed by centrifuging in a benchtop centrifuge (5 min, max speed, 4 °C). Supernatants were used for enzyme activity determination.

For total RNA isolation, cells were washed twice with PBS and collected in 350 µL RLT buffer (Qiagen, Hilden, Germany) supplemented with 1% *β*-mercaptoethanol according to manufacturer’s protocol. Samples were stored at ‒80 °C until further analysis.

### Immunoblot analysis

Aliquots of cell lysates (20 µg protein per lane) were analyzed by SDS-PAGE on 12% Tris–glycine minigels, and transferred onto Immobilon-P membranes (Millipore, Billerica, MA, USA). Primary and secondary antibodies were applied overnight at 4 °C and for 1 h at room temperature, respectively. Horseradish peroxidase (HRP)-conjugated goat polyclonal anti-Actin (Cell Signaling, Danvers, MA, USA, sc-1616) antibodies were used at 1:2000 dilution. SCD1 was detected with a rabbit polyclonal antibody (Cell Signaling, Danvers, MA, USA, 2438S), used at a dilution of 1:2000, followed by HRP-conjugated goat polyclonal anti-rabbit IgG (Cell Signaling, Danvers, MA, USA, 7074S) at a dilution of 1:2000. ETS1 was detected with a goat polyclonal antibody (Bethyl Laboratories, A190-110A), used at a dilution of 1:2000, followed by HRP conjugated mouse monoclonal anti-goat IgG (Cell Signaling, Danvers, MA, USA, sc-2354) at a dilution of 1:2000. HRP was detected by C-DiGit® Blot Scanner (LI-COR, Lincoln, NE, USA) using the SuperSignalWest Pico Chemiluminescent Substrate (Thermo Fisher Scientific,Waltham, MA, USA). As the edges of the membranes can blend into the background due to digital imaging, a protein marker is run on each side of the sample sets to clearly define them. Uncropped versions of all parallel blot images are available in the Supplementary Information file.

### Luciferase assay

Luciferase activity was detected using the Luciferase Assay System kit (Promega, Madison, WI, USA) by adding 15 µL Luciferin reagent to 5 µL of al cell extracts. *β*-galactosidase activity of 20 µL cell lysates was measured by determining the *o*-nitrophenyl-*β*-D-galactopyranoside (at a final concentration of 3 mM) cleavage rate. Luminescence was detected using a Varioskan multi-well plate reader (Thermo Fisher Scientific, Waltham, Massachusetts, USA). Values for luciferase activity were normalized to *β*-galactosidase activity (measured by standard protocol using the same Varioskan plate reader in photometry mode). Each experiment was repeated three times independently, and each sample was analyzed in triplicate.

### RNA isolation, cDNA synthesis

Total RNA was purified from transfected HEK293T and HepG2 cells by using RNeasy Plus Mini Kit (Qiagen, Germantown, MD, USA) following the manufacturer’s instruction. Concentrations were measured using NanoDrop1000 spectrophotometer. To assess the integrity and purity of the isolated total mRNA samples, the ratios of their absorbance at 260/280 and 260/220 nm were determined, and they were also analyzed by agarose gel electrophoresis to visualize bands corresponding to 28S and 18S rRNAs, respectively. Possible DNA contamination was removed by DNase I treatment using RNAqueous®-4PCR Kit (Invitrogen, Carlsbad, CA, USA). cDNA samples were produced by reverse transcription of 0.5 µg DNA-free RNA, using the SensiFAST™ cDNA Synthesis Kit (Meridian Bioscience, Memphis, TN, USA).

#### qPCR

Quantitative PCR assay was performed in 20 µL final volume containing 5 µL 20 × diluted cDNA, 1 × PowerUp™ SYBR™ Green Master Mix, and 0.5 µM forward and reverse primers using QuantStudio 12 K Flex Real-Time PCR System (Thermo Fisher Scientific, Waltham, Massachusetts, USA). *SCD1* and *ETS1* sequences were amplified by 5’‒ CTG GCC TAT GAC CGG AAG AAA ‒3’ / 5’‒ GAC CCC AAA CTC ATT CCA TAG G ‒3’ and 5’ – AGA TGA GGT GGC CAG GAG AT ‒ 3’ / 5’ – CTG CAG GTC ACA CAC AAA GC – 3’ primer pairs, respectively. *GAPDH* cDNA was also amplified as an endogenous control using 5’ ‒ GTC CAC TGG CGT CTT CAC CA ‒ 3’ / 5’ ‒ GTG GCA GTG ATG GCA TGG AC ‒ 3’ primer pair. The first step of the thermocycle was an initial denaturation and enzyme activation at 95 °C for 2 min. It was followed by 40 cycles of 95 °C for 15 s, 55 °C for 15 s, and 72 °C for 1 min; measurement of the fluorescent signal was carried out during annealing. Reactions were performed in triplicates, and a reaction mixture with RNase-free water instead of template cDNA was employed as non-template control. Relative expression levels were calculated as 2^‒*Δ*CT^, where *Δ*C_T_ values corresponded to the difference of the C_T_-values of the endogenous control and target genes.

#### Subjects

282 patients diagnosed with T2DM in the 2nd Department of Internal Medicine, Semmelweis University (51.2% female, 48.8% male, disease onset at the age of 62.4 ± 12.6 y) were recruited in the study. The control group consisted of 370 volunteers with no medical history of any metabolic disease (61.4% female, 38.6% male, mean age: 33.1 ± 21.6 y). The diagnosis of diabetes was made based on fasting blood sugar values, oral glucose tolerance test (OGTT), and HbA_1C_ value according to WHO regulations. Individuals with autoimmune, infectious, or metabolic disorders other than type 2 diabetes were excluded from the study. Genetic analysis of the participants was approved by the Local Ethical Committee (ETTTUKEB ad.328/KO/2005, ad.323–86/2005-1018EKU from the Scientific and Research Ethics Committee of the Medical Research Council). The study was conducted in accordance with the principles of the Declaration of Helsinki. Participants signed a written informed consent before sample collection for genetic analysis. To avoid the risk of spurious association caused by population stratification, subjects of Hungarian origin were exclusively included to ensure the comparison of homogenous populations. Buccal epithelial cells were collected by swabs. The first step of DNA isolation was an incubation of the buccal samples at 56 °C overnight in 0.2 mg/mL Proteinase K cell lysis buffer. Subsequently, proteins were denatured using a saturated NaCl solution. DNA was then precipitated by isopropanol and 70% ethanol. DNA pellet was resuspended in 100 µL 0.5 × TE (1 × TE: 10 mM Tris pH = 8.0; 1 mM EDTA) buffer. Concentration of the samples was measured by NanoDrop1000 spectrophotometer.

#### Genotyping

Rs1054411 promoter polymorphism of the *SCD1* gene was genotyped using pre-designed TaqMan assay (C_34192814_10, Thermo Fisher Scientific, Waltham, MA, USA). qPCR assay was performed in 5 µL final volume containing approximately 4 ng genomic DNA, 1 × TaqPath™ ProAmp™ Master Mix, and 1 × TaqMan® SNP Genotyping Assay using QuantStudio 12 K Flex Real-Time PCR System (Thermo Fisher Scientific, Waltham, MA, USA). Thermocycle was started by activating the hot start DNA polymerase and denaturing genomic DNA at 95 °C for 10 min. This was followed by 40 cycles of denaturation at 95 °C for 15 s, and combined annealing and extension at 60 °C for 1 min. Real-time detection was carried out during the latter step to verify the results of the subsequent post-PCR plate reads and automatic genotype calls.

#### Statistical analysis

Immunoblots were evaluated by densitometry using the Image Studio® 5.2 software (LI-COR Biotechnology, Lincoln, NE, USA), and are shown as relative band densities normalized to Actin as a reference. Relative band densities, luciferase activities and mRNA levels are presented in the diagrams as mean values ± S.D. and were compared by ANOVA with the Tukey’s multiple comparison post hoc test, using the GraphPad Prism 6.0 software (GraphPad Software, Boston, MA, USA). Differences with a *p* < 0.05 value were considered to be statistically significant. Genotype–phenotype association was assessed by *χ*^2^-test comparing the genotype distribution of the patient and the control groups (i.e., additive model). Power of the genetic association study was assessed by the GAS Power Calculator on line tool (https://csg.sph.umich.edu/abecasis/cats/gas_power_calculator/) using the additive disease model (prevalence of T2DM is 6.28%, genotype relative risk was 1.2.).

### Supplementary Information

Supplementary Information.

## Data Availability

All data are available in the main text or in the supplementary material. The raw data and uncropped blot images underlying the above presented results, as well as all Supplementary Figures and Tables are enclosed in the Supplementary Information file. Any additional data from this study is available from the corresponding authors (zambo.veronika@med.semmelweis-univ.hu and kereszturi.eva@semmelweis.hu) upon reasonable request.
