# Supplementary material for: Allele-specific effect of various dietary fatty acids and ETS1 transcription factor on SCD1 expression
Source: Sci Rep. 2024 Jan 2;14:177. doi: 10.1038/s41598-023-50700-5 (PMC10761808; doi:10.1038/s41598-023-50700-5)

SUPPLEMENTARY INFORMATION

for

Allele-specific effect of various dietary fatty acids and ETS1 transcription factor on SCD1 expression

Kinga Tibori[1,#], Veronika Zámbó[1,#,*], Gabriella Orosz[1], Péter Szelényi[1], Farkas Sarnyai[1], Viola Tamási[1], Zsolt Rónai[1], Miklós Csala[1] and Éva Kereszturi[1,*]

[1] Department of Molecular Biology, Semmelweis University, H-1085 Budapest, Hungary

[*] Corresponding authors: Veronika Zámbó: zambo.veronika@med.semmelweis-univ.hu

Éva Kereszturi: kereszturi.eva@semmelweis.hu

[#] These authors contributed equally to this work and share first authorship.

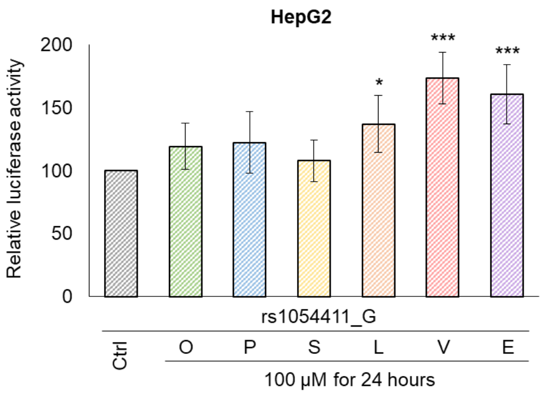

Supplementary Figure S1. ***Modulating effect of the rs1054411_G variant on SCD1 promoter activity in the presence of various FAs in HepG2 cells.*** Transient transfection and FA treatment of HepG2 cells were performed as described in *Materials and Methods*. pCMV-β-gal vector served as transfection control. Luciferase and β-galactosidase enzyme activities were measured as indicated in *Materials and Methods* and their relative ratios are shown as bar graphs. The diagram depicts the results of six independent measurements normalized to the relative luciferase activity of wild type *SCD1* promoter containing reporter vector. Data are shown as mean values ± S.D. Statistical analysis was performed by using the Tukey-Kramer Multiple Comparisons Test. Ctrl: control; O: oleate; P: palmitate; S: stearate; L: linoleate; V: vaccenate; E: elaidate; *$p < 0.05$; *** $p < 0.001$.

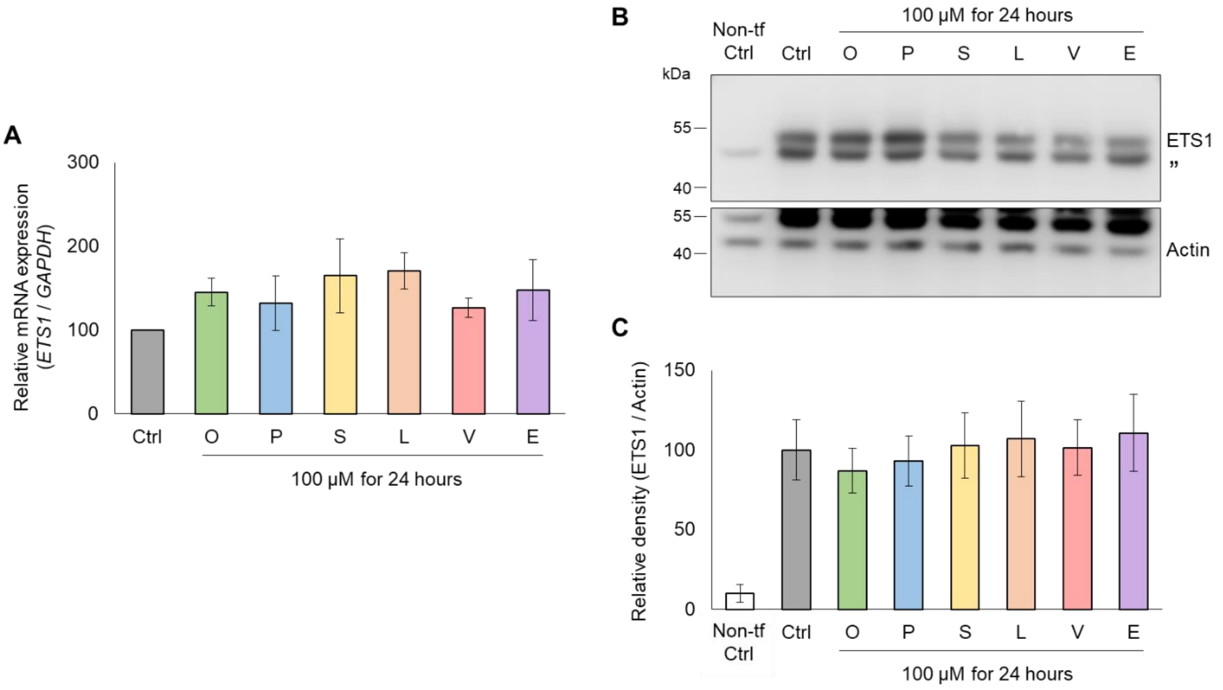

Supplementary Figure S2. *ETS1 mRNA and protein expression in FA-treated HEK293T cells.* (***A***) The levels of endogenous *ETS1* mRNA were measured in HEK293T cells after FA-treatment as described in *Materials and Methods*. qPCR was carried out using *ETS1* and *GAPDH* sequence specific primers as indicated in *Materials and Methods*. (***B***) Immunoblot analysis of ETS1 transfected and FA-treated samples (20 µg protein per lane) was carried out using anti-ETS1 and anti-Actin antibodies as described in *Materials and Methods*. The band intensities were determined by densitometry and ETS1/Actin ratios are shown as bar graphs (***C***). Representative results of six independent experiments are presented. Uncropped versions of all parallel blot images are available in the Supplementary Information file. Statistical analysis was performed with the Tukey-Kramer Multiple Comparisons Test. Data are shown as mean values ± S.D. Non-tf Ctrl: non-transfected control; Ctrl: control; O: oleate; P: palmitate; S: stearate; L: linoleate; V: vaccenate; E: elaidate.

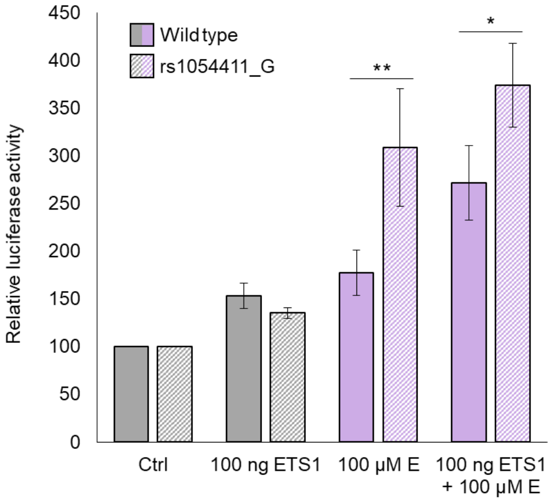

Supplementary Figure S3. ***Combined effect of elaidate and ETS1 TF on the transcriptional activity of wild type and rs1054411_G allele-containing SCD1 promoter construct.*** Transient co-transfection and elaidate treatment of HEK293T cells were performed as described in *Materials and Methods*. pCMV-*β*-gal vector served as transfection control. Luciferase and *β*-galactosidase enzyme activities were measured as indicated in *Materials and Methods* and their relative ratios are shown as bar graphs. The diagram depicts the results of three independent measurements normalized to ETS1-free and FA-untreated wild type or rs1054411_G pGL3-SCD1 promoter vector, respectively. Data are shown as mean values ± S.D. Statistical analysis was performed by using the Tukey-Kramer Multiple Comparisons Test. Ctrl: control; E: elaidate; $^{*}p < 0.05$; $^{**}p < 0.01$.

Supplementary Table S1. ***SNPs in SCD1 promoter.*** The position of the polymorphisms was calculated upstream from the ATG sequence of the start codon. MAF: minor allele frequency based on 1000Genomes Project global frequency data; VEP: Variant Effect Predictor

SNP ID	Position	Alleles		MAF (%)	VEP
		wt	mut		
**rs1054411**	–11	C	G	28	modifier
**rs670213**	–895	C	T	33	modifier
**rs2275657**	–964	G	C	44	modifier
**rs2275656**	–1057	G	C	45	modifier

Supplementary Table S2. ***List of transcription factors that are affected by rs670213, rs2275657 or rs2275656 polymorphisms.*** Positive or negative values of the relative score differences indicate that the minor allele increases or decreases the probability of TF binding, respectively.

SNP ID	Name	TF ID	Strand	Relative score (%)		
				wt allele	mut allele	Difference
**rs670213**	TFAP2A	MA0003.1	+	92.89	73.99	–18.90
	RHOXF1	MA0719.1	+	80.65	63.97	–16.68
**rs2275657**	NFATC3	MA0625.2	–	64.77	83.72	18.94
	USF1	MA0093.1	–	70.16	85.55	15.39
**rs2275656**	TFAP2A	MA0003.1	+	78.11	97.01	18.90
	NR2C2	MA1536.1	–	64.15	80.59	16.43
	NR5A1	MA1540.1	–	77.70	93.60	15.89
	PITX2	MA1547.2	–	80.83	65.76	–15.07
	TFE3	MA0831.1	–	82.07	65.90	–16.17
	RHOXF1	MA0719.1	–	80.74	64.06	–16.68
	TFAP2A	MA0003.1	–	88.76	69.86	–18.90

Supplementary Table S3. ***Sequence and annealing temperature of mutagenic primers.***

Nucleotide exchanges are indicated by underlined, bold lowercase letters.

Mutagenic primer name	Sequence 5' → 3'	T_m (°C)
rs1054411C>G-S	TCCCGGCATC**g**GAGAGCCAAG	68
rs1054411C>G-AS	TCACTTTCCAGGGGGCTG	68
rs670213T>C-S	GCGTACCGAG**c**CCCCCGCGCT	72
rs670213T>C-AS	CTGCGAACAATGGCTCTGCCCC	72
rs2275657G>C-S	GCCGGAGTCC**c**GTGCGGTCCC	72
rs2275657G>C-AS	CGCACACACAGGCTGGCTG	72
rs2275656G>C-S	GGAGGCGCGG**c**CTTGGGGATG	71
rs2275656G>C-AS	AGGATGCGTGCGGGATGTTTTG	71

RAW DATA AND UNCROPPED PARALLEL BLOT IMAGES

Raw data from the parallel measurements for Figure 2.

		Raw data from parallel measurements					
		**1**	**2**	**3**	**4**	**5**	**6**
**HEK293T**	Control	1.0000	1.0000	1.0000	1.0000	1.0000	1.0000
	Oleate	0.4816	0.5355	0.4750	0.6316	0.6023	0.4997
	Palmitate	0.7722	0.9500	0.8403	1.3739	1.1331	1.1391
	Stearate	0.7900	0.8562	0.7600	1.1804	1.0070	0.9989
	Linoleate	0.4850	0.4810	0.4320	0.6387	0.4987	0.3392
	Vaccenate	0.5407	0.5712	0.5602	0.6931	0.6093	0.6775
	Elaidate	1.1266	1.1290	1.0681	1.1626	1.1574	1.1357
**HepG2**	Control	1.0000	1.0000	1.0000	1.0000	1.0000	1.0000
	Oleate	0.7485	0.7615	0.7022	0.9733	1.1065	1.0725
	Palmitate	1.0133	1.2016	0.8907	0.8760	1.0928	1.0578
	Stearate	0.7584	0.9164	0.7331	1.0381	1.1204	1.0688
	Linoleate	0.5052	0.5363	0.4497	0.6792	0.6741	0.6151
	Vaccenate	0.8491	0.9599	0.7802	1.0042	0.9808	0.8963
	Elaidate	1.3698	1.4990	1.2570	1.5911	1.8075	1.4682

Raw data from the parallel measurements for Figure 3 and Figure S1.

		Raw data from parallel measurements					
		**1**	**2**	**3**	**4**	**5**	**6**
**HEK293T_wild type**	pGL3B	4.5208	3.5446	5.4969			
	Ctrl	100	100	100			
	Oleate	87.5456	70.8595	98.1963			
	Palmitate	176.3224	169.3916	159.8816			
	Stearate	210.2666	175.2895	157.0359			
	Linoleate	59.1908	60.8540	65.9066			
	Vaccenate	84.0760	83.0323	98.4538			
	Elaidate	164.3835	162.5544	142.8906			
**HepG2_wild type**	pGL3B	5.6724	5.1636	2.3207	7.7344	5.4071	5.2643
	Ctrl	100	100	100	100	100	100
	Oleate	124.8208	118.4468	87.7268	103.2309	114.3045	93.2777
	Palmitate	167.2374	148.1188	156.1893	116.8043	150.0352	147.7361
	Stearate	109.7015	109.9472	87.3507	153.8701	165.5364	142.7141
	Linoleate	65.8446	65.8953	76.5431	57.9766	66.0873	75.8792
	Vaccenate	105.7482	108.1154	106.1017	118.6211	121.6350	116.0605
	Elaidate	141.4078	145.2770	142.8981	140.2837	157.5402	151.6285
**HepG2_rs1054411_G**	pGL3B	4.5208	3.5446	5.4969	2.5685	6.4731	4.1954
	Ctrl	100	100	100	100	100	100
	Oleate	177.1071	129.2955	109.3192	142.6296	119.6027	90.9347
	Palmitate	264.2045	194.2415	155.6990	165.2122	151.8526	152.8599
	Stearate	118.1038	90.6968	83.4301	193.1272	200.8175	165.2437
	Linoleate	96.1405	85.8130	111.4598	80.9084	108.6677	73.7777
	Vaccenate	185.4981	154.5052	189.9238	207.7129	250.8584	190.6803
	Elaidate	280.3364	232.9681	193.9307	244.3086	250.5832	206.8983

Raw data from the parallel measurements for Figure 4.

		Raw data from parallel measurements		
		**1**	**2**	**3**
**HEK293T**	pGL3B	1.4575	1.4930	1.6391
	wild type	100	100	100
	rs1054411_G	103.5124	106.5186	118.2009
	rs670213_T	107.1035	103.3534	104.5392
	rs2275657_C	97.8929	119.6090	125.2989
	rs2275656_C	100.2991	97.0993	98.3805
**HepG2**	pGL3B	1.0258	1.1388	1.0834
	wild type	100	100	100
	rs1054411_G	117.4904	110.7000	111.1400
	rs670213_T	119.8236	120.4381	109.0063
	rs2275657_C	104.1043	112.2283	124.6635
	rs2275656_C	119.8135	133.0622	98.6209

Raw data from the parallel measurements for Figure 5.

		Raw data from parallel measurements				
		1	2	3	4	5
Oleate	wild type	100	100	100		
	rs1054411_G	266.0754	212.9208	127.7334		
	rs670213_T	135.0339	121.9017	105.3650		
	rs2275657_C	181.9737	164.2025	110.7993		
	rs2275656_C	96.0981	90.3058	61.9995		
Palmitate	wild type	100	100	100		
	rs1054411_G	221.4435	208.4599	192.6623		
	rs670213_T	185.4433	160.5381	135.7835		
	rs2275657_C	188.3156	197.8056	176.7981		
	rs2275656_C	142.5280	120.5159	105.3305		
Stearate	wild type	100	100	100		
	rs1054411_G	131.7549	187.7334	182.5707		
	rs670213_T	119.6696	153.3618	136.2453		
	rs2275657_C	134.7414	176.0477	157.6633		
	rs2275656_C	101.9167	100.8172	85.8222		
Linoleate	wild type	100	100	100		
	rs1054411_G	121.9888	184.8278	165.6482		
	rs670213_T	112.4320	135.6726	124.3802		
	rs2275657_C	113.7269	157.9097	126.4075		
	rs2275656_C	111.5822	122.9289	93.8648		
Vaccenate	wild type	100	100	100	100	100
	rs1054411_G	221.9245	167.2575	153.1748	233.2481	204.7510
	rs670213_T	151.2630	117.9171	126.2069	150.9382	143.1189
	rs2275657_C	219.2379	171.0909	128.3702	163.8137	196.4445
	rs2275656_C	94.7502	82.6531	82.0032	95.4816	80.9867
Elaidate	wild type	100	100	100	100	100
	rs1054411_G	314.8614	220.1760	198.6909	303.2081	350.6967
	rs670213_T	163.8228	108.2451	107.7099	134.7395	204.9394
	rs2275657_C	202.1486	166.3732	139.3967	236.8190	245.6827
	rs2275656_C	125.4626	83.4499	69.9385	77.3882	146.4093

Raw data from the parallel measurements for Figure 6.

Raw data from parallel measurements							
		Ctrl	co-transfected with ETS1 (ng)				
			10	25	50	100	200
wild type	**1**	100	117.5436	115.2941	137.3195	164.5061	210.9642
	**2**	100	141.8488	135.0137	147.9700	185.6236	251.0331
	**3**	100	127.1147	132.2091	131.0399	176.0003	267.5602
	**4**	100		126.3775	140.0938	179.8128	199.8951
	**5**	100		160.7026	170.9713	184.5789	227.3386
	**6**	100		162.7077	166.5789	171.5266	232.7068
	**7**	100		135.2701	153.2323	218.0663	206.8646
	**8**	100		147.5997	175.9603	217.0663	232.3300
	**9**	100		154.4408	181.2328	207.0810	225.9488
	**10**	100		148.4729	145.3859	247.2123	
	**11**	100		157.3396	181.2277	227.7618	
	**12**	100		146.7683	194.2332	186.0079	
rs1054411_G	**1**	100	92.1093	115.4607	122.8088	162.0167	172.5478
	**2**	100	124.0591	127.4530	129.5998	183.5608	190.5310
	**3**	100	118.7126	108.4746	147.0621	136.6598	176.8736
	**4**	100		104.0015	141.6435	124.0852	214.4109
	**5**	100		126.9319	141.8914	131.8609	165.3647
	**6**	100		133.7630	137.1363	152.3895	204.8627
	**7**	100		123.5579	143.4272	165.4645	203.0888
	**8**	100		114.6007	161.1879	155.0487	213.3302
	**9**	100		147.0508	150.7448	184.4912	214.0472
	**10**	100		80.5644	118.4594	153.3537	
	**11**	100		108.1177	114.5153	170.6240	
	**12**	100		113.9783	121.0002	196.2531	

Raw data from the parallel measurements for Figure S2.

		Raw data from parallel measurements					
		1	2	3	4	5	6
HEK293T	Control	1.0000	1.0000	1.0000	1.0000	1.0000	1.0000
	Oleate	1.6010	1.0410	1.4980	1.6888	1.0483	1.8429
	Palmitate	2.1615	0.3877	0.9266	1.7183	1.0563	1.6737
	Stearate	3.2648	0.9869	1.5326	2.2815	1.0246	1.4064
	Linoleate	2.5919	1.7183	1.8519	1.7303	1.2861	1.6381
	Vaccenate	1.4520	1.2058	1.2226	1.4773	0.8503	1.3861
	Elaidate	2.6009	0.9653	0.7341	1.8251	0.9164	1.8100

Raw data from the parallel measurements for Figure S3.

		Raw data from parallel measurements		
		1	2	3
wild type	Ctrl	100	100	100
	100 ng ETS1	168.3734	149.0137	142.2091
	100 μM E	176.4076	202.0227	154.2855
	100 ng ETS1 + 100 μM E	254.5030	316.0191	243.2500
rs1054411_G	Ctrl	100	100	100
	100 ng ETS1	130.0140	134.2002	141.3065
	100 μM E	351.9041	238.4530	335.2284
	100 ng ETS1 + 100 μM E	369.8339	419.2328	332.0416

SCD1 protein expression in HEK293T cells treated with 100 μM FAs for 24h

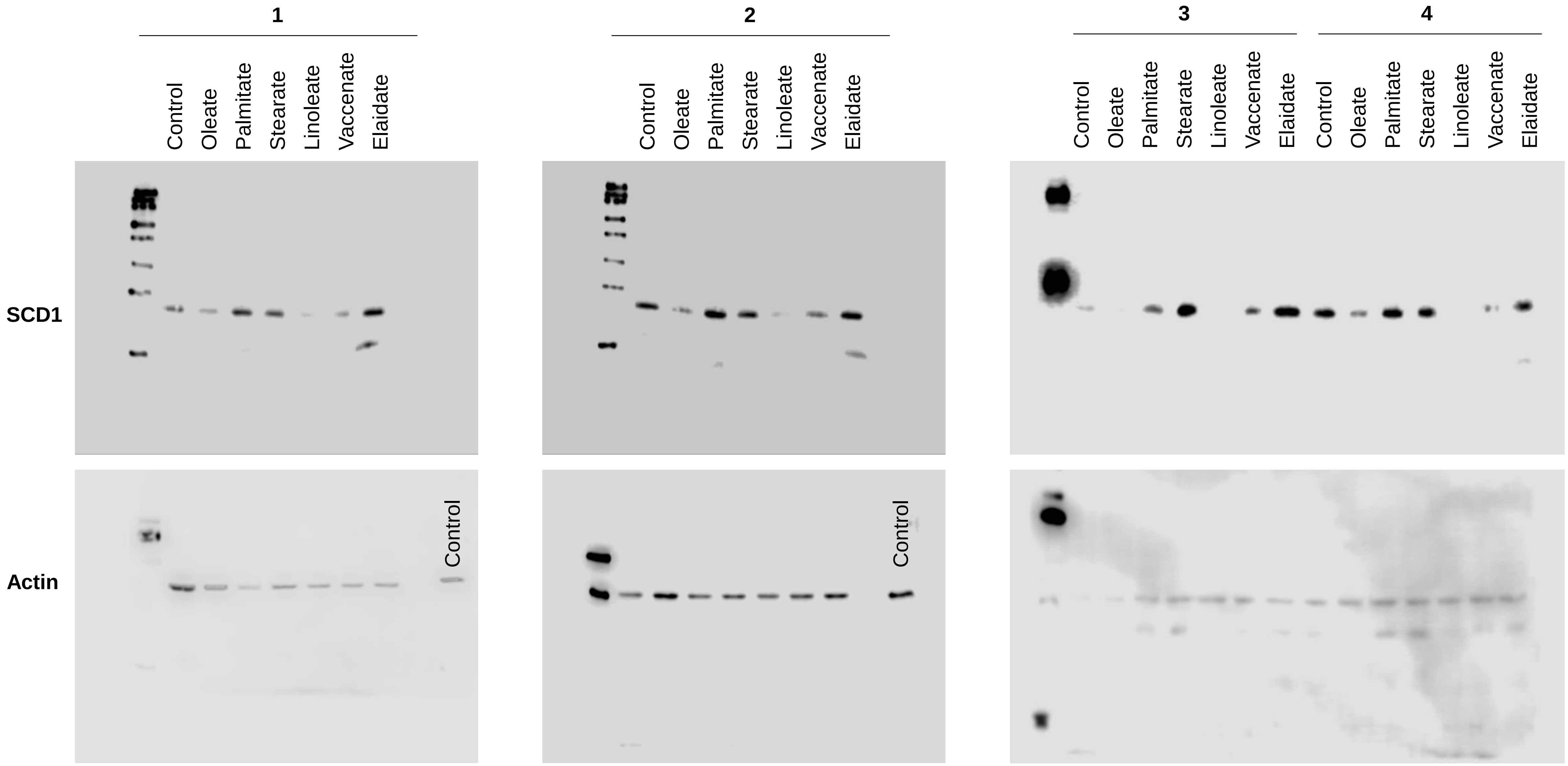

SCD1 protein expression in HepG2 cells treated with 100 µM FAs for 24h I.

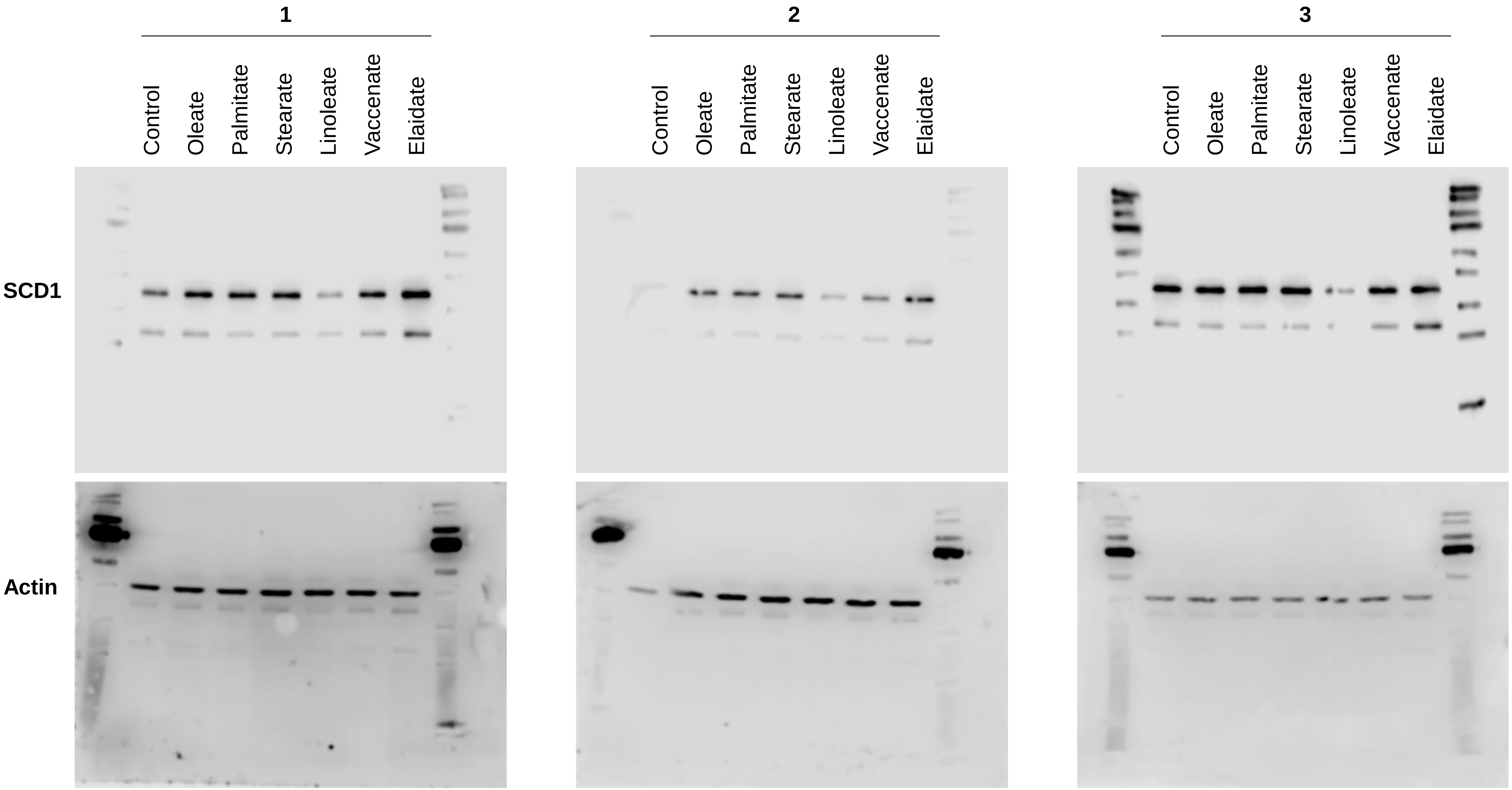

SCD1 protein expression in HepG2 cells treated with 100 µM FAs for 24h II.

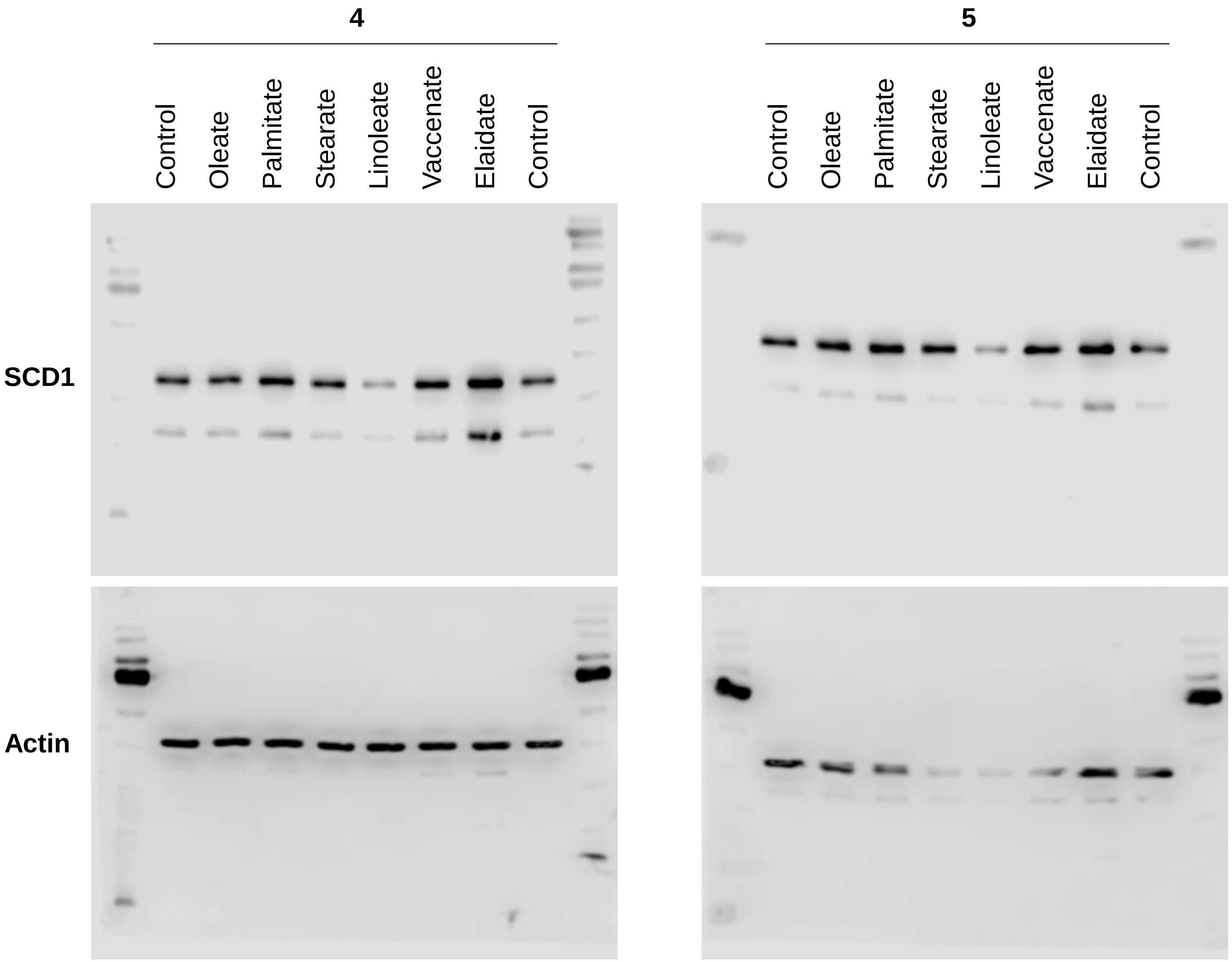

ETS1 protein expression in transfected HEK293T cells treated with 100 µM FAs for 24h

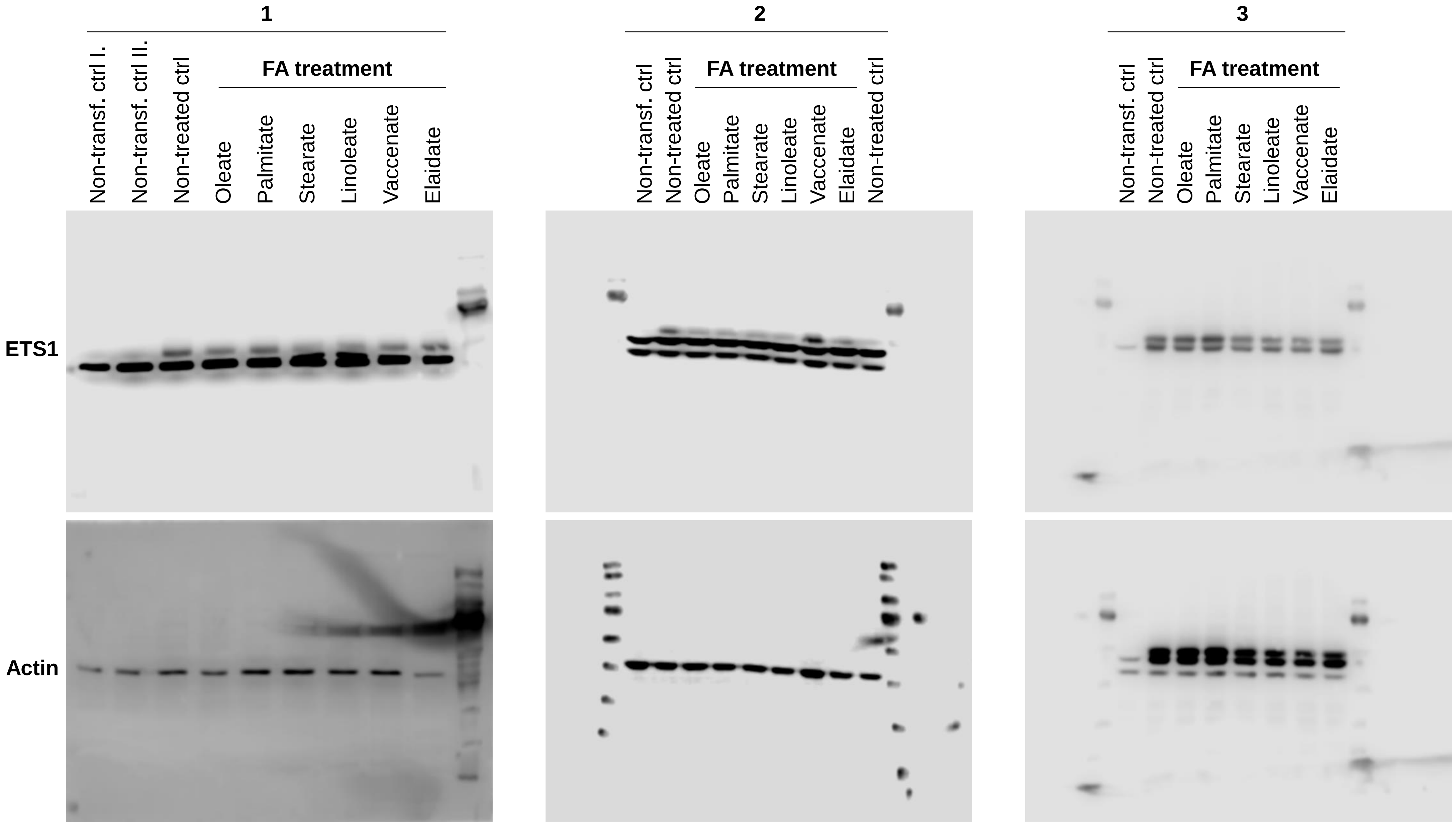

ETS1 protein expression in HEK293T cells transfected with increasing amounts of ETS1 plasmid

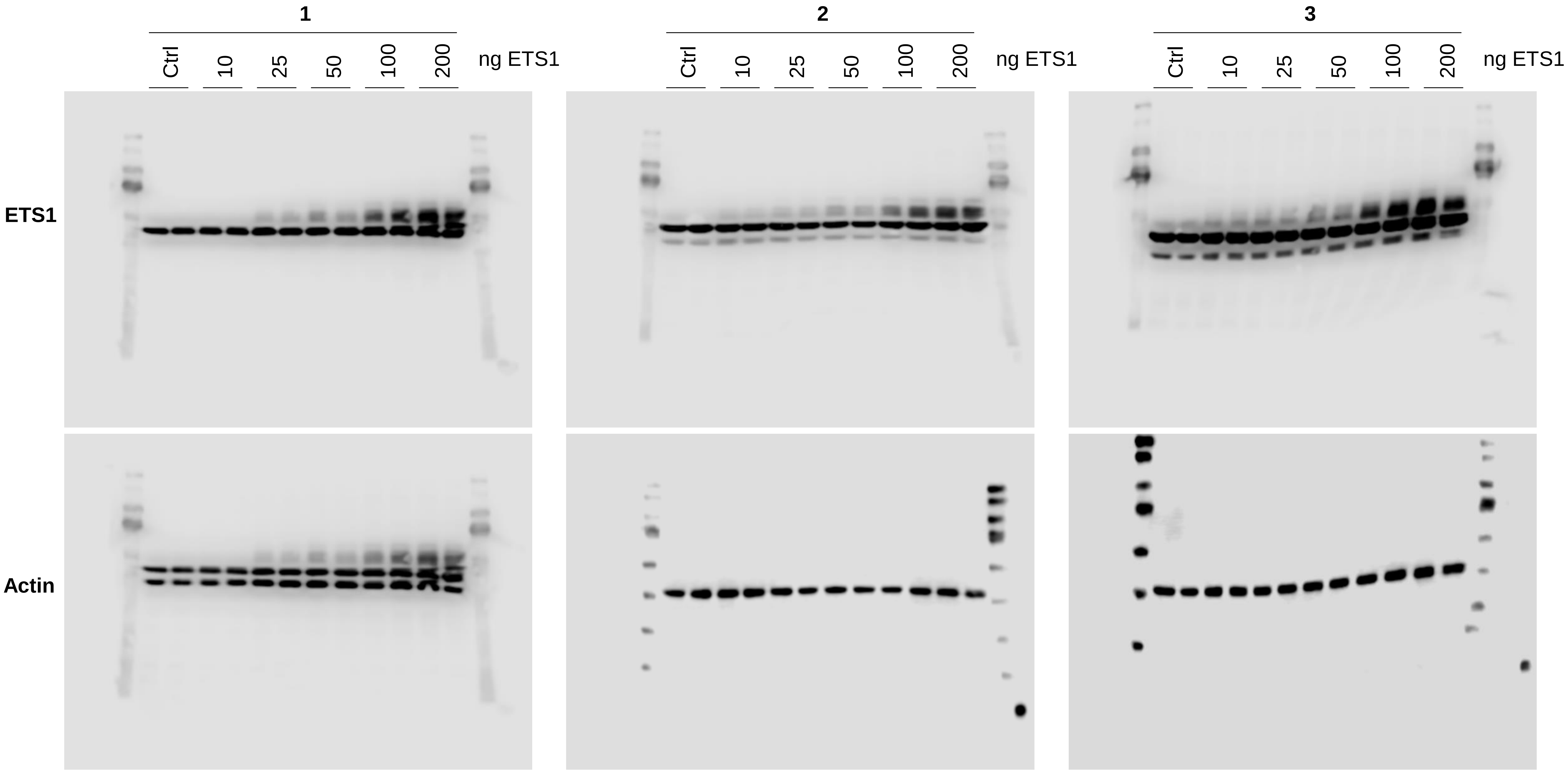

Supplement: Supplementary file 1 — Supplementary Information. [file 41598_2023_50700_MOESM1_ESM.pdf]
